# Potential benefits of oral administration of *AMORPHOPHALLUS KONJAC* glycosylceramides on skin health – a randomized clinical study

**DOI:** 10.1186/s12906-019-2721-3

**Published:** 2020-01-31

**Authors:** Sudeep Heggar Venkataramana, Naveen Puttaswamy, Shyamprasad Kodimule

**Affiliations:** Research Scientist, No. 14A, KIADB, R & D Center for Excellence, Vidya Herbs (P) Ltd., Jigani Industrial Area, Bangalore, Anekal Taluk 560 105 India

**Keywords:** Phytoceramides, Skin barrier function, Human clinical

## Abstract

**Background:**

Ceramides play a fundamental role in maintaining the skin health as a function of improved barrier permeability. Reduced ceramide content results in skin dryness and wrinkledness. Intake of dietary ceramides potentially compensates the skin ceramide content. In the present study we have assessed the skin health benefits of oral supplementation of a hydroalcoholic extract from *Amorphophallus konjac* tubers standardized to 5% glycosylceramides, in a placebo-controlled clinical trial.

**Methods:**

Fifty-one healthy human volunteers (aged 18–60 years) were supplemented with 100 mg/day of either a placebo or *A. konjac* extract capsules (5 mg glycosylceramides) for 6-weeks. The skin parameters were evaluated through dermatological diagnosis. Subject perceived efficacy of the product was further evaluated by a self-assessment questionnaire.

**Results:**

Oral intake of *A. konjac* extract significantly decreased the skin dryness, hyperpigmentation, redness, itching and oilyness (*p* < 0.05). The improvement in skin health following intake of *A. konjac* extract was observed to be time-dependent from the start. Further, *A. konjac* extract was well-tolerated throughout the study, as no adverse events or toxic changes were recorded.

**Conclusion:**

The study demonstrates the skincare properties of orally ingested glycosyl ceramides from konjac tubers.

**Trial registration:**

CTRI/2018/12/016661 dated 13/12/2018 retrospectively registered, http://ctri.nic.in/Clinicaltrials/showallp.php?mid1=19851&EncHid=&userName=SkinCera

## Background

Ceramides are a variety of sphingolipids, present in the skin keeping it moist and healthy [1]. Human epidermis is composed of the stratum basale, stratum spinosum, granulosa, and stratum corneum from the inner layer towards outside. In the stratum basale, phospholipids and cholesterol are the main components while granulosa majorly contains glycosylceramides. Ceramide in the stratum corneum plays a key role in barrier functions such as moisture retention, maintenance of skin architecture, prevention of invasion of foreign bodies [2, 3].

During the recent past, studies have demonstrated the beneficial effects of orally supplemented ceramides against dry skin, skin aspect, and associated discomforts. Animal studies have been conducted to describe the oral bioavailability of ceramide. Dietary glycosylceramides are metabolized in rat small intestine and found in portal blood after hydrolysis by ceramidases in the gastrointestinal tract [4]. Nevertheless, a large proportion of ingested sphingolipids are excreted in the feces, animal studies suggest that after oral intake radiolabeled ceramides are metabolized, absorbed and distributed to many tissues, including the skin [5]. Ueda et al., showed that orally administrated radiolabeled D2-sphingosine is transferred to the skin, from dermis to epidermis in an unchanged structural form, and further generates radiolabeled glucosylceramides and ceramides by in vivo biosynthesis in mice [6]. The orally ingested sphingolipids in human beings could be hypothesized to follow a similar track of metabolism as described in animal studies. Not all the ingested sphingolipids are hydrolyzed and absorbed. However, some components of sphingolipids (sphingosine) reach the systemic circulation following transport through the mucosa. The dietary ceramides may activate the ceramide synthesis in the skin improving the barrier function, rather than direct reutilization reaching the skin [7]. In addition, previous studies have demonstrated that oral supplementation with ceramides may be beneficial for skin permeability barrier homeostasis and parameters such as hydration and/or barrier function, elasticity, and recovery after induced disruption of barrier dysfunction [8–10].

Loss of ceramides causes dry skin and dermatitis that subsequently leads to the appearance of wrinkle in the skin [11]. Supplementation of ceramides thus is essential for maintaining skin barrier permeability and hydration. Nevertheless, synthetic ceramides are used in general as cosmetics; natural ceramides have drawn much attention during the recent past due to the safety [12]. Plant derived ceramides are chemically identical to those found in our skin. Currently there are several types of ceramides available in the market commercially derived from plant sources such as rice, wheat, soy, and spinach. Glycosylceramides have been reported to improve skin barrier function in hairless mice by intake of extract of food materials containing glycosylceramide. Oral intake of glycosylceramide reduces transepidermal water loss in normal adult or in atopic dermatitis patients [13].

*Amorphophallus konjac* (Family: Araceae) is a perennial plant commonly known as konjak or konnyaku. Konjac is a traditional food ingredient and medicine used in China, Japan and South East Asi*a. konjac* is used in the Chinese folk medicine as a tumour suppressor, detoxification and phlegm liquefaction. *A. konjac* is a rich source of glycosyl ceramides and glucomannan. Konjac tuber-derived glycosylceramides have been commercially exploited as dietary supplements for dry skin [14]. Previous studies in mice have shown that konjac glycosylceramides are effective against trans-epidermal water loss [9]. Usuki et al. [14] have reported the efficacy of konjac ceramides on itch-causing neurite outgrowth in PC12 cells [15]. Based on the experimental and clinical evidences from previous studies we have evaluated the effects of orally ingested konjac extract containing glycosylceramides on skin care properties in a placebo-controlled, randomized, single-blind clinical trial with healthy human volunteers.

## Methods

### Investigational product

This study was conducted to investigate the efficacy of SkinCera™, a proprietary extract from the tubers of *A. konjac*. The formal identification of the plant material was done at Vidya Japan K.K., Minato-ku, Tokyo, Japan. SkinCera is a hydroalcoholic extract standardized to 5% of glycosyl ceramides.

### Trial design

This was a monocentric single-blinded, placebo-controlled, randomized study performed on 50 healthy human volunteers. The study was conducted in compliance with the protocol, International Conference on Harmonization (ICH) Good Clinical Practice (GCP) Guidelines, including ICH E6, and applicable local regulatory requirements and laws. This clinical trial adheres to the CONSORT guidelines. The information on the nature, purpose, and risks of the study were provided to each subject or subject’s legally authorized representative before their participation. Written informed consent for participation and publication of the data was obtained prior to the subject entering the study (before initiation of protocol-specified procedures). The subjects were randomized to two intervention groups: placebo and *A. konjac* extract (capsule form, 100 mg/day).

### Ethics, consent and permissions

The study protocol and informed consent form were approved by institutional ethics committee (SKAMC/96/2017–18, Sri Kalabyraveshwara Swamy Ayurvedic Medical College, Hospital and Research Center, India). This clinical study was registered in Clinical Trials Registry – India (CTRI/2018/12/016661). Informed consent and consent to publish were obtained prior to subject randomization. Subjects meeting all inclusion and no exclusion criteria signed a written informed consent and enrolled in the study.

### Participants

#### Eligibility criteria

Healthy adult male and female volunteers aged 18–60 years presenting symptoms such as skin dryness, roughness, itching, redness, hyperpigmentation, blackheads and whiteheads were recruited. Subjects with chronic medical disorders were excluded from the study. Other exclusion criteria were pregnant or lactating women, smokers and alcoholics, subjects undergoing other cosmetic treatments. Further, subjects unwilling/unable to comply with the protocol requirements were excluded from the study.

### Study site

The study was performed at Sri Kalabyraveshwara Swamy Ayurvedic Medical College, Hospital and Research Center, Bangalore, India.

### Interventions

**Fig. 1:**
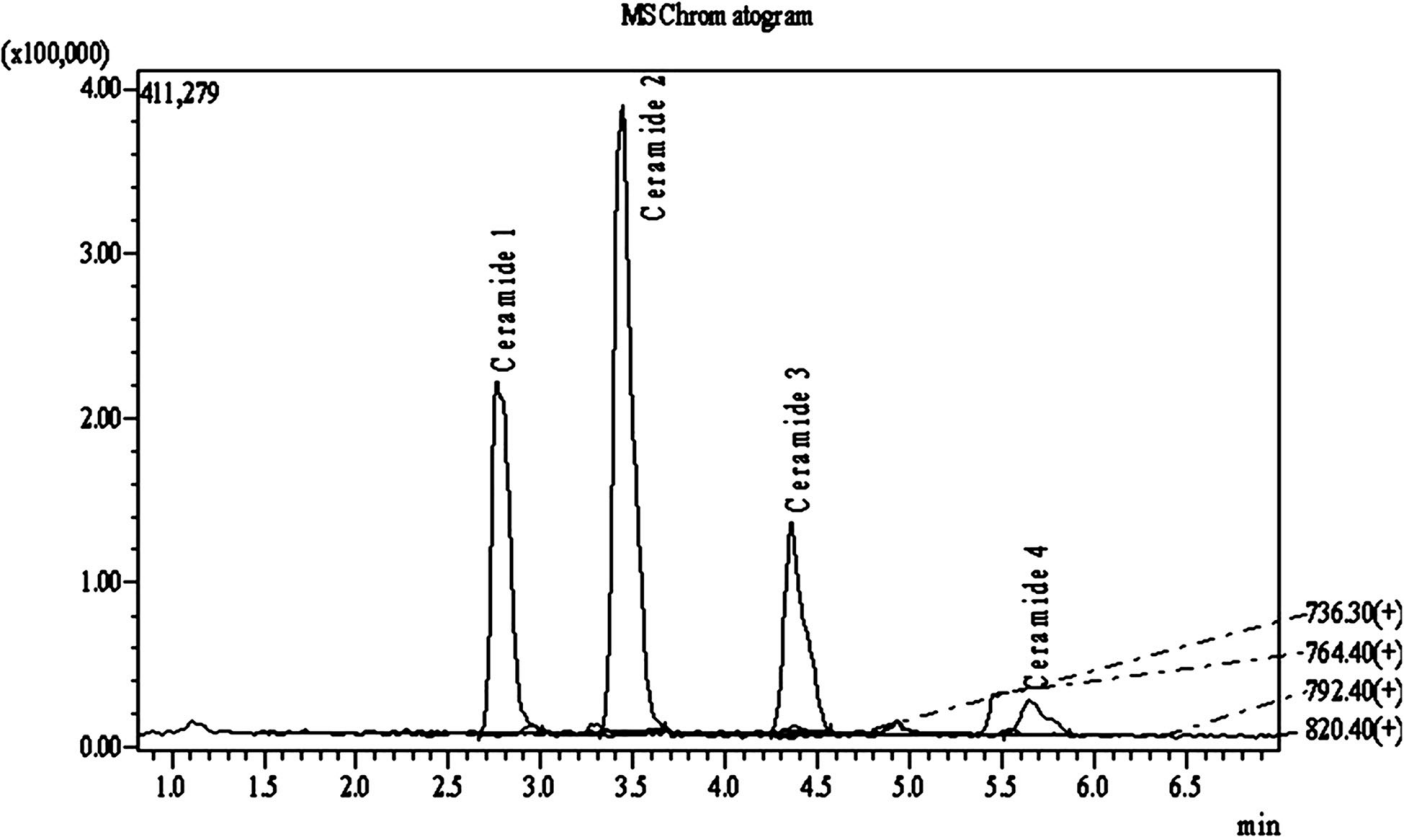
Analysis of glycosylceramides in SkinCera by LCMS/MS

A standardized extract from *A. konjac* tubers was prepared according to a proprietary manufacturing process using solvent based extraction strategy. The raw material was procured from Gunma prefecture, Japan and authenticated internally at Vidya Herbs Pvt. Ltd., Japan. *A. konjac* extract is standardized to ≥5% glycosylceramides (ceramide 1–4, Fig. 1) by LCMS/MS analysis. Further details on the constituents of *A. konjac* extract are provided in supplementary file **(**Additional file 3**)**.

During the 6-week treatment period, the daily oral intake was two capsules containing either placebo (maltodextrin) or *A. konjac* extract (=100 mg/day). Indeed, SkinCera group received 5 mg/day of glycosylceramides. All capsules were of the same appearance, color and odor. Table 1 shows the composition of capsules. The subjects had to record their daily food consumption during the intervention period to ensure that there was no change in the dietary habits throughout study period.

**Table 1:** Quantitative formula of treatments (per capsule)

Composition	SkinCera (mg)	Placebo (mg)
Maltodextrin	70	120
*A. konjac* extract	50	0
Dicalcium phosphate	100	100
Magnesium carbonate	50	50
Magnesium stereate	5	5
Silica micronized	25	25
Total weight	300	300

### Outcomes

The primary end point was to assess the effect of *A. konjac* extract consumption on skin health. The efficacy evaluation was performed after a regular interval of 3 weeks of ingestion: Visit 1 (day 1), visit 2 (third week of treatment) and visit 3 (after 6 weeks of treatment). The skin parameters included in the study were evaluated as overall symptoms through dermatological diagnosis and inquiry. Diagnosis score is usually a number that conveys the response of a subject to the treatment (Additional file 2). With reference to the previously validated subjective scoring scales, the diagnosis score test was developed by the investigator before the conduct of study [16, 17]. In each evaluation, anchor points were prepared and evaluated by the investigator. A total of eight skin parameters were considered for the study and the subjects were evaluated by the investigator at every scheduled visit. The safety analysis was summarized for vital signs and adverse event monitoring.

The subject perception of the product efficacy was evaluated by a validated self-assessment questionnaire at follow-up visit.

### Sample size

The sample size calculation was based on the expected difference between mean scores of the two treatments considered to be medically relevant. Assuming a common standard deviation of 1.8 for the number of assessment parameters at the end of treatment, 20 per group would be enough to detect a difference of 1.63 in mean score difference between the two treatments with power of 80% and a 0.05 two-sided level of significance. Considering a dropout rate of 10% the sample size was finalized as 50 (25 per group). The details of the sample size calculation are presented as supplementary file **(**Additional file 4**)**.

### Randomization and blinding

The subjects were randomly assigned to the study groups in a 1:1 ratio to receive active treatment and placebo. Block randomization was used to assign the subjects to treatment groups. Each randomized subject received a xx-digit randomization number. Randomized subjects who terminated their study participation for any reason, regardless of whether the IP was taken or not, retained their randomization number. Subjects were kept blind to treatment group assignment.

### Statistical analysis

The primary analysis was based on the full analysis set which included all randomized subjects and followed the intent-to-treat principle. Statistical analysis was performed using SPSS software version 16.0. All statistical significance tests were 2-sided and performed at the 5% significance level. Data were analyzed by one-way ANOVA followed by Dunnet test.

## Results

### Subject enrollment and baseline data

**Fig. 2:**
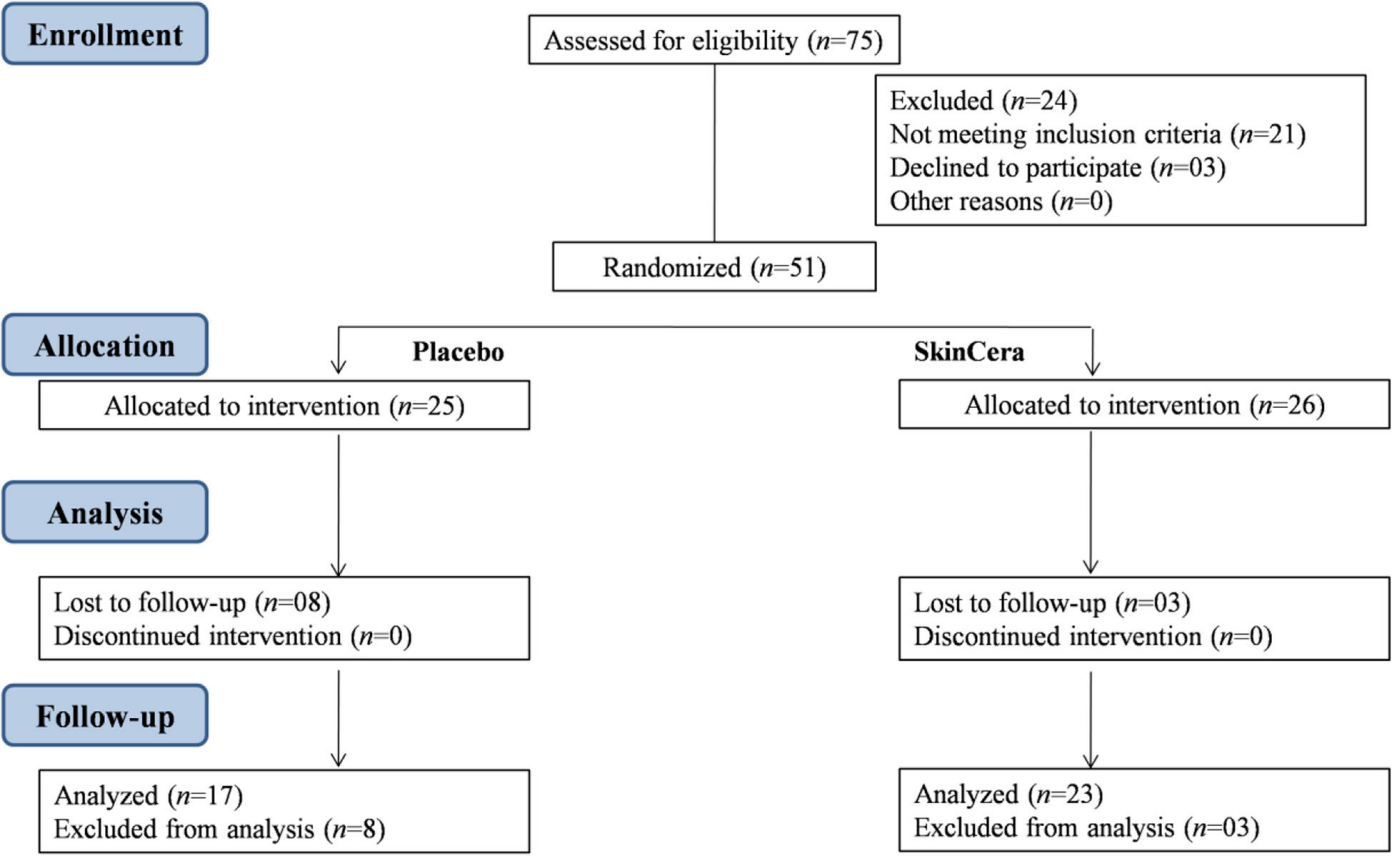
Subject participation flow chart

A total of 75 subjects were screened from single study center and 51 volunteers were enrolled into the study. Out of these 51 subjects, 40 subjects completed the study and 11 were withdrawn from the study due to lost to follow-up/failed to return (Fig. 2). Subject baseline demographic data are presented in Table 2. Further details are provided in Additional file 1.

**Table 2:** Baseline demographics of subjects

	SkinCera	Placebo
Number of subjects	26	25
Age (years)	28.08 ± 7.3	32.56 ± 5.3
Height	163.2 ± 10.3	165.8 ± 7.27
Weight	62.4 ± 14.63	65.9 ± 6.82

The data were statistically analyzed at *p* < 0.05 and represented as mean ± SD

### Dermatological diagnosis

**Fig. 3:**
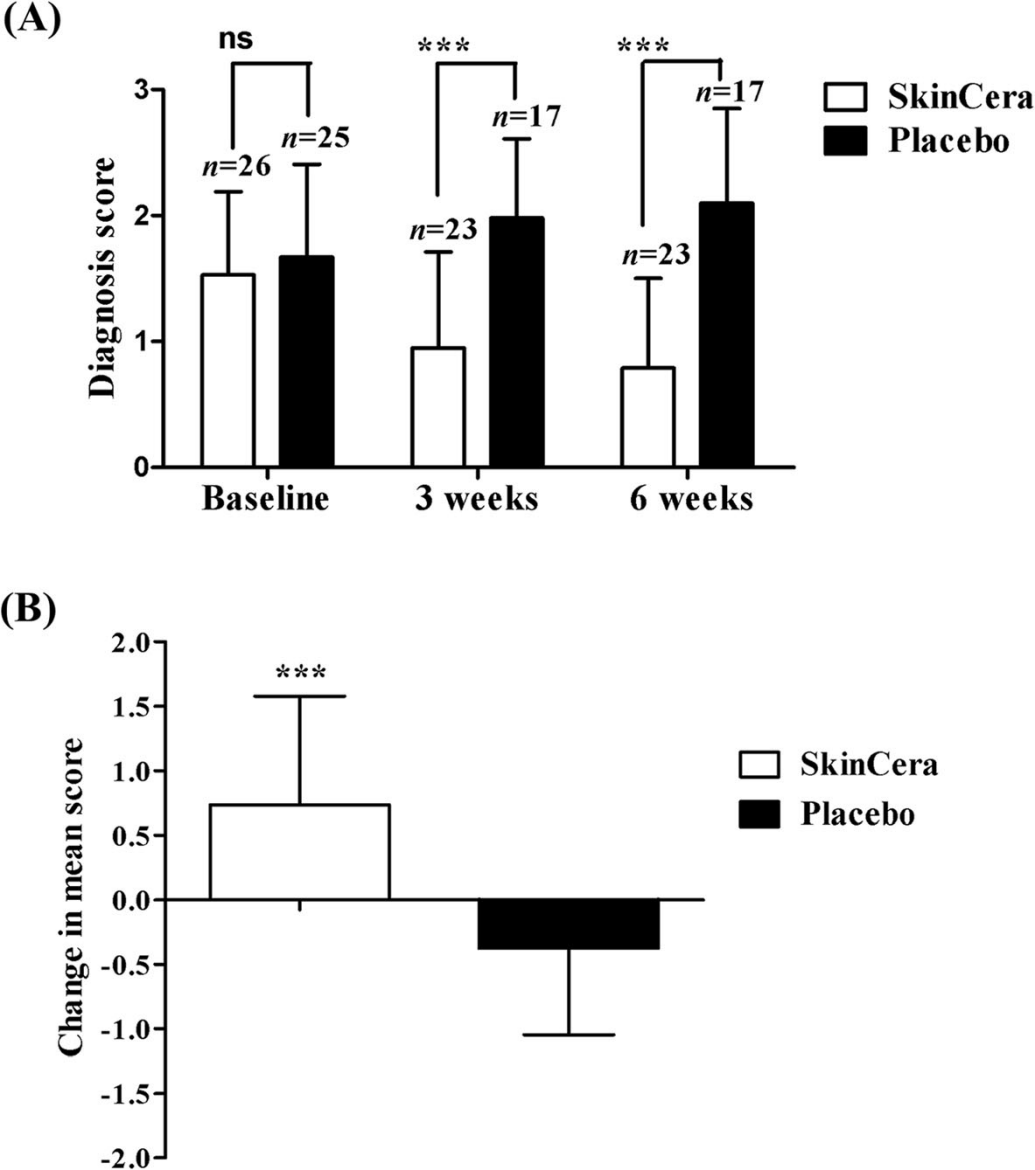
Effect of SkinCera on dermatological diagnosis score. **a** Mean diagnosis score at baseline, 3 weeks and 6 weeks after SkinCera intake (**b**) Change in mean score from baseline to end of study. Values are expressed as mean ± SD. The data were analyzed by one-way ANOVA followed by Dunnet’s t test. ***p < 0.001 compared to placebo. ns, not significant

The mean dermatological diagnosis score, before and after ingestion of *A. konjac* extract is presented in Table 3. In *A. konjac* extract treated group, the skin dryness was evident among 8 subjects with a mean severity symptom score of 1.88 at the screening visit (before treatment). The subjects showed significant improvement during the study having a mean score of 0.63 after 6 weeks treatment (*p* < 0.01). Similarly, *A. konjac* extract treatment markedly decreased the mean score of hyperpigmentation from 1.58 at baseline to 1.053 after the 6-week treatment (*p* < 0.05). Skin redness was observed initially among ten subjects in the *A. konjac* extract group with a mean score of 1.7. The subject response to skin redness, itching and oilyness following *A. konjac* extract treatment were significant at *p* < 0.05 after 6 weeks. *A. konjac* extract treatment was appreciable for skin roughness though the data were not significant. Subject response in the *A. konjac* extract group for other skin parameters such as whiteheads/blackheads and lesions were moderate. It was further observed that the study parameters were aggravated among the subjects in the placebo group during the study. The overall mean diagnosis score decreased significantly (*p* < 0.001) after six-week oral ingestion of *A. konjac* extract (Fig. 3).

**Table 3:** Dermatological diagnosis before and after 6-week treatment with SkinCera

	SkinCera group				Placebo group			
Parameter	Number of subjects with symptoms	Before treatment	After 3 weeks	After 6 weeks	Number of subjects with symptoms	Before treatment	After 3 weeks	After 6 weeks
Dryness	8	1.88 ± 0.99	0.5 ± 0.75**	0.63 ± 0.51**	12	1.33 ± 0.49	1.58 ± 0.51	1.67 ± 0.65
Whiteheads/ blackheads	10	1.56 ± 0.72	1.0 ± 0.86	1.11 ± 0.78	11	1.91 ± 0.83	2.46 ± 0.52	2.55 ± 0.69
Hyperpigmentation	21	1.47 ± 0.77	1.05 ± 0.52	1.05 ± 0.40*	11	2.46 ± 0.52	2.36 ± 0.5	2.14 ± 0.69
Redness	10	1.7 ± 0.67	1.2 ± 0.91	0.6 ± 0.84*	5	1.20 ± 0.44	1.8 ± 0.44	LTF
Lesions	11	1.3 ± 0.48	1.1 ± 0.87	0.7 ± 0.82	5	1.4 ± 0.54	1.8 ± 0.45	LTF
Itching	10	1.67 ± 0.5	0.88 ± 0.78	0.67 ± 0.86*	3	1.33 ± 0.57	2.33 ± 0.57	LTF
Oilyness	12	1.46 ± 0.52	0.90 ± 0.83	0.73 ± 0.78*	9	1.11 ± 0.33	1.22 ± 0.44	1.71 ± 0.48*
Roughness	7	1.29 ± 0.48	0.71 ± 0.75	0.57 ± 0.78	11	1.91 ± 0.83	2.27 ± 0.47	2.36 ± 0.81
Overall	26	1.53 ± 0.66	0.95 ± 0.76***	0.79 ± 0.71***	25	1.67 ± 0.74	1.98 ± 0.63*	2.10 ± 0.75**

The values are the mean in each group; analyzed by one-way ANOVA followed by Dunnet’s t test. The data were statistically significant at *p < 0.05, **p < 0.01, ***p < 0.001 within the group. LTF, Lost to follow-up

0 (no symptoms) - 1 (mild) - 2 (moderate) - 3 (severe)

**Fig. 4:**
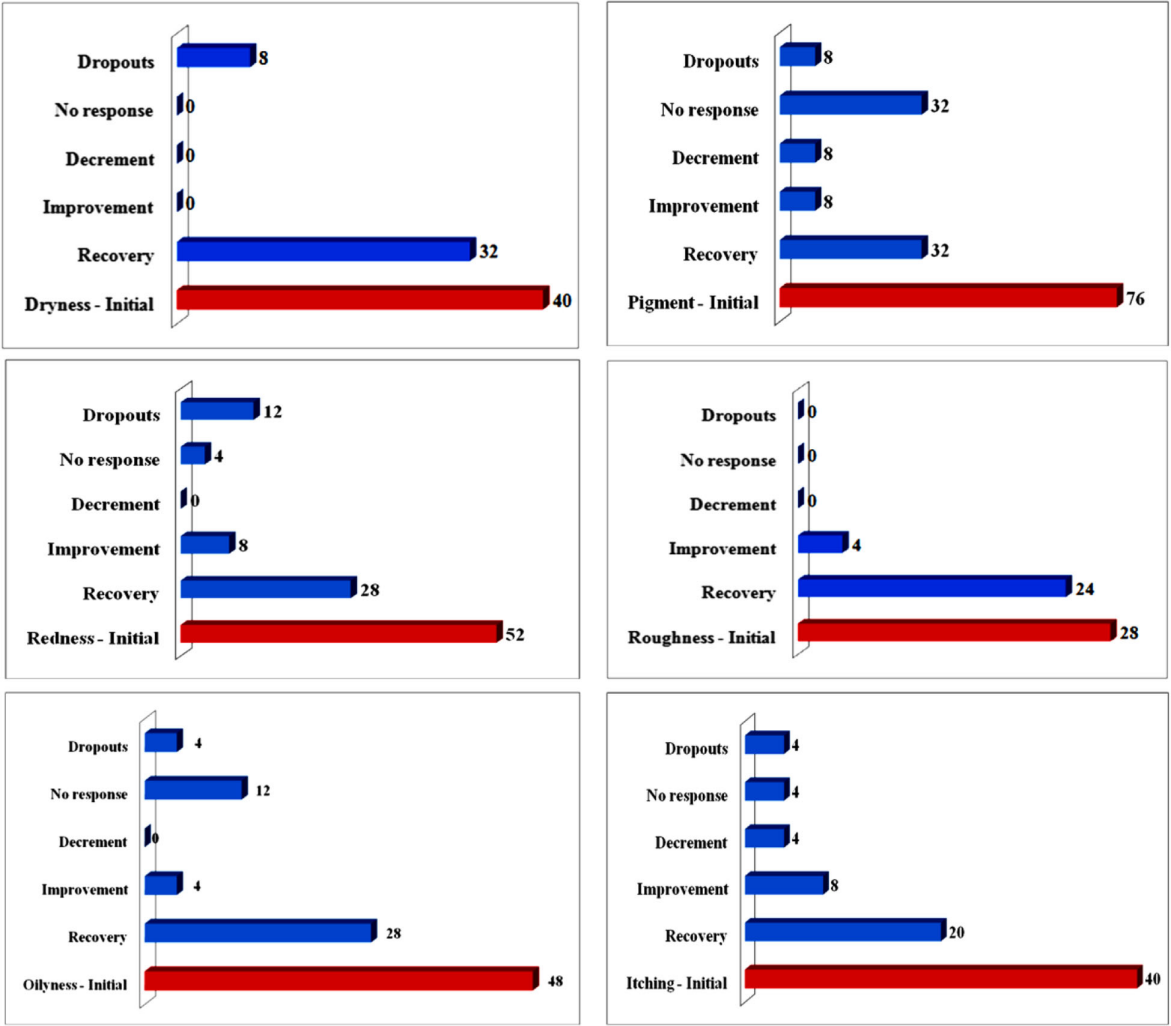
Subject response (%) to SkinCera treatment for individual skin parameters (at follow-up visit)

**Fig. 5:**
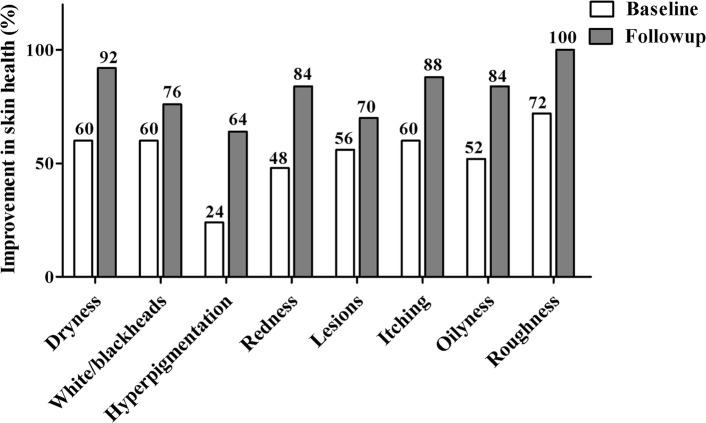
Effect of SkinCera on the improvement in skin health from baseline to follow-up

The percentage response of subjects following 6-week treatment with 100 mg/day treatment with *A. konjac* extract, to individual skin parameters is presented in Fig. 4**.** The recovery percentage of subjects from skin dryness and roughness were highly appreciable among the various parameters assessed. Figure 5 shows the percentage improvement in the subject population following *A. konjac* extract treatment. *A. konjac* extract considerably alleviated the skin condition as evident from the increase in the percentage of subjects showing improvement for respective parameters.

### Adverse events

*A. konjac* extract treatment did not induce any significant adverse or serious adverse effects throughout the study period as evident from the vital signs and health monitoring (Data not shown).

### Results of self-assessment questionnaire

The subjects’ perception as evaluated through self-assessment questionnaire was consistent with the results of the diagnosis. Results of the self-assessment questionnaire are presented in Table 4.

**Table 4:** Results of self-assessment questionnaire after 6-week of supplementation with SkinCera

Questions and self assessment	SkinCera	Placebo
How do you rate the efficacy of the product in skin dryness improvement?		
Insufficient	0%	65%
Sufficient	30%	30%
Fairly good	35%	5%
Excellent	35	0%
Do you think your skin texture is improved?		
Yes	75%	30%
No	20%	60%
No opinion	5%	10%
How do you evaluate the product efficacy concerning improvement in skin pigmentation?		
Insufficient	20%	75%
Sufficient	30%	20%
Fairly good	45%	5%
Excellent	5%	0%
Do you think your skin is smoother and softer?		
Yes	80%	20%
No	15%	65%
No opinion	5%	15%

## Discussion

Ceramides from edible plants have emerged as a safe and preferred alternative to animal ceramides for cosmetic applications [18]. Research on several plant-based ceramides are intended to supplement effective amount of the ceramide content to the skin which otherwise has reduced content of natural ceramides due to defective skin barrier function [9, 19]. Human clinical trials on ceramide-based products from various plant sources like wheat, rice, beet and konjac tubers have been documented to improve the skin barrier functions [8, 10, 20]. The clinical evidence on the efficacy of konjac glycosylceramides in other skincare properties apart from skin dryness is lacking. This clinical trial was undertaken to study the effect of supplementation of small quantity of glycosylceramides (5 mg/day) from konjac on overall skin health benefits in healthy human volunteers. A six-week ingestion of *A. konjac* extract at 100 mg/day (glycosylceramide, 5 mg/day) significantly improved the skin parameters such as dryness, hyperpigmentation, itching and oilyness compared to placebo group. Interestingly, these beneficial effects were evident after 3-weeks of supplementation and continued over the entire study period. It is assumed that the ingested sphingolipids converted to sphingolipid metabolites which are absorbed through the intestine and distribute in the blood. Subsequently these metabolites reach the skin and play their physiological role to restore skin dryness [20].

In the present study a scoring scale for the diagnosis of the symptom severity was used to assess the efficacy of *A. konjac* extract. As a preliminary clinical trial this study included only the subjective measurement. Subjective scores are validated and clearly reflect the symptom severity. Even though the extent and intensity of skin condition are central to measure the severity, the symptoms of patients are subjective; hence an ideal scoring system should include the subjective symptoms [21, 22]. The overall efficacy of *A. konjac* extract was found to be increasing significantly with time compared to the baseline. Our results agreed with the subject perception of the product efficacy as determined through the self-assessment questionnaire. Importantly, oral intake of *A. konjac* extract did not induce any toxic signs and was well tolerated throughout the study period. The present study, however, lacks the performance data collected via objective assessment.

## Conclusion

In this clinical trial, we have shown that daily oral intake of *A. konjac* extract (glycosylceramides) significantly improved the skin health compared to placebo. However, considering the limitations of study being single-blinded and subjective, further trials are required using a double-blinded study design including the objective measurements to further explore the efficacy of *A. konjac* extract, and its effect on the enhancement of ceramide content in the skin.

## Supplementary information

**Additional file 1.** Additional data. Characterization of SkinCera, Study event schedule, subject disposition, subject demographic data analysis, effect of SkinCera treatment on percentage subject response to skin parameters, analysis of vital signs and diagnosis scoring scale.

**Additional file 2.** Questionnaire. Diagnosis questionnaire including the symptom scoring scale.

**Additional file 3.** Characterization of constituents in SkinCera. Details of other constituents of SkinCera apart from glycosylceramides 1–4.

**Additional file 4.** Sample size calculation.

## Data Availability

The data sets used and/or analysed during the current study available from the corresponding author on reasonable request.

## Abbreviations

GCP: Good Clinical Practice
ICH: International Conference on Harmonization
LCMS/MS: Liquid chromatography-Mass Spectrometry
