# Supplementary material for: Potential benefits of oral administration of AMORPHOPHALLUS KONJAC glycosylceramides on skin health – a randomized clinical study
Source: BMC Complement Med Ther. 2020 Jan 31;20:26. doi: 10.1186/s12906-019-2721-3 (PMC7076855; doi:10.1186/s12906-019-2721-3)
Supplement: Supplementary file 1 — Additional file 1. Additional data. Characterization of SkinCera, Study event schedule, subject disposition, subject demographic data analysis, effect of SkinCera treatment on percentage subject response to skin parameters, analysis of vital signs and diagnosis scoring scale. [file 12906_2019_2721_MOESM1_ESM.docx]

**Additional data**

**Table 1**. Study events schedule

|  | **Screening** | **Visit 1** | **Visit 2** | **Visit 3** | **Follow-up visit** |
| --- | --- | --- | --- | --- | --- |
| Informed consent process | X |  |  |  |  |
| Medical and surgical history | X |  |  |  |  |
| Prior medications history | X |  |  |  |  |
| Demographic data | X |  |  |  |  |
| Physical examination (including vital signs) | X | X | X | X | X |
| Review of I/E criteria | X | X |  |  |  |
| Randomization |  | X |  |  |  |
| Dispensing medications |  | X | X |  |  |
| Skin examination | X | X | X | X | X |
| AE assessment |  |  | X | X | X |
| Review of concomitant medications |  | X | X | X | X |
| Review of subject diary (compliance, AEs, Con meds and dietary details) |  |  | X | X | X |
| Dispensing of subject diary |  | X | X | X |  |
| Treatment compliance |  |  |  | X |  |

**Table 2**. Disposition of subjects

|  | **SkinCera (N=26)** | | **Placebo (N=25)** | | **All (N=51)** | |
| --- | --- | --- | --- | --- | --- | --- |
|  | **n** | **%** | **n** | **%** | **All** | **%** |
| No. of patients enrolled | 26 | 100 | 25 | 100 | 51 | 100 |
| No. of patients completed | 23 | 88.5 | 17 | 68 | 40 | 78.4 |
| No. of patients withdrawn | 03 | 11.5 | 08 | 32 | 11 | 21.57 |
| Reason for withdrawal | 0 | 0.00 | 0 | 0.00 | - | - |

**Table 3.** Number of subjects at each visit

|  | **SkinCera (N=26)** | | **Placebo (N=25)** | | **All (N=51)** | |
| --- | --- | --- | --- | --- | --- | --- |
| Visit | **n** | **%** | **n** | **%** | **All** | **%** |
| Visit 1 | 26 | 100 | 25 | 100 | 51 | 100 |
| Visit 2 | 23 | 88.5 | 25 | 100 | 48 | 94.1 |
| Visit 3 | 23 | 88.5 | 17 | 68.0 | 40 | 78.4 |
| Follow-up visit | 23 | 88.5 | 0 | 0.00 | 23 | 45.1 |

**Table 4A**. Summary of subject gender at baseline (Intention-to-Treat population)

|  | **SkinCera (N=26)** | | **Placebo (N=25)** | | **All (N=51)** | |
| --- | --- | --- | --- | --- | --- | --- |
| **Gender** | **n** | **%** | **n** | **n** | **%** | **n** |
| Male | 5 | 19.23 | 9 | 36.0 | 14 | 27.45 |
| Female | 19 | 80.76 | 16 | 64.0 | 35 | 68.62 |

**Table 4B**. Summary of subject demographic characteristics at baseline

(Intention-to-Treat population)

|  | **SkinCera (N=26)** | **Placebo (N=25)** | **All (N=51)** |
| --- | --- | --- | --- |
| **Age (Years)** | | | |
| N | 26 | 25 | 51 |
| Mean | 28.08 | 32.56 | 30.27 |
| SD | 7.3 | 5.3 | 6.7 |
| Minimum | 18 | 25 | 18 |
| Median | 25.5 | 32 | 30 |
| Maximum | 48 | 45 | 48 |
| **Height (cm)** | | | |
| N | 26 | 25 | 51 |
| Mean | 163.2 | 165.8 | 164.5 |
| SD | 10.3 | 7.27 | 8.95 |
| Minimum | 142 | 158 | 142 |
| Median | 162 | 163 | 163 |
| Maximum | 186 | 180 | 186 |
| **Weight (kg)** | | | |
| N | 26 | 25 | 51 |
| Mean | 62.42 | 65.9 | 64.13 |
| SD | 14.63 | 6.82 | 11.5 |
| Minimum | 40 | 57 | 40 |
| Median | 59 | 63.8 | 63 |
| Maximum | 101 | 78 | 101 |

**Table 5**. Effect of SkinCera treatment on the percentage subject response to skin dryness

|  | **Subject visits** | | | | |
| --- | --- | --- | --- | --- | --- |
| **Response** | **Screening** | **Day 1** | **3 weeks** | **6 weeks** | **Follow-up** |
| Dryness | 40 | 40 | - | - | - |
| Recovery | - | - | 20 | 12 | **32** |
| Improvement | - | - | 8 | 20 | - |
| Decrement | - | - | - | - | - |
| No response | - | - | 4 | 4 | - |
| Drop outs | - | - | 8 | 8 | 8 |

**Table 6**. Effect of SkinCera treatment on the percentage subject response to skin white/black heads

|  | **Subject visits** | | | | |
| --- | --- | --- | --- | --- | --- |
| **Response** | **Screening** | **Day 1** | **3 weeks** | **6 weeks** | **Follow-up** |
| Black/white heads | 40 | 40 | - | - | - |
| Recovery | - | - | 8 | 4 | **8** |
| Improvement | - | - | 8 | 8 | **8** |
| Decrement | - | - | - | 4 | - |
| No response | - | - | 20 | 20 | 20 |
| Drop outs | - | - | 4 | 4 | 4 |

**Table 7.** Effect of SkinCera treatment on the percentage subject response to skin hyperpigmentation

|  | **Subject visits** | | | | |
| --- | --- | --- | --- | --- | --- |
| **Response** | **Screening** | **Day 1** | **3 weeks** | **6 weeks** | **Follow-up** |
| Pigment | 84 | 76 | - | - | - |
| Recovery | - | - | 4 | 8 | **32** |
| Improvement | - | 8 | 16 | 12 | **8** |
| Decrement | - | - | - | 4 | 8 |
| No response | - | - | 56 | 52 | 32 |
| Drop outs | - | - | 8 | 8 | 8 |

**Table 8**. Effect of SkinCera treatment on the percentage subject response to skin redness

|  | **Subject visits** | | | | |
| --- | --- | --- | --- | --- | --- |
| **Response** | **Screening** | **Day 1** | **3 weeks** | **6 weeks** | **Follow-up** |
| Redness | 52 | 52 | - | - | - |
| Recovery | - | - | 8 | 20 | **28** |
| Improvement | - | - | 8 | 8 | **8** |
| Decrement | - | - | - | 4 | - |
| No response | - | - | 24 | 8 | 4 |
| Drop outs | - | - | 12 | 12 | 12 |

**Table 9**. Effect of SkinCera treatment on the percentage subject response to skin lesions

|  | **Subject visits** | | | | |
| --- | --- | --- | --- | --- | --- |
| **Response** | **Screening** | **Day 1** | **3 weeks** | **6 weeks** | **Follow-up** |
| Lesions | 44 | 44 | - | - | - |
| Recovery | - | - | 12 | 20 | **16** |
| Improvement | - | - | - | 4 | **4** |
| Decrement | - | - | 4 | - | 12 |
| No response | - | - | 24 | 16 | 8 |
| Dropouts | - | - | 4 | 4 | 4 |

**Table 10**. Effect of SkinCera treatment on the percentage subject response to skin itching

|  | **Subject visits** | | | | |
| --- | --- | --- | --- | --- | --- |
| **Response** | **Screening** | **Day 1** | **3 weeks** | **6 weeks** | **Follow-up** |
| Itching | 40 | 40 | - | - | - |
| Recovery | - | - | 12 | 20 | **20** |
| Improvement | - | - | 12 | 4 | **8** |
| Decrement | - | - | - | 8 | 4 |
| No response | - | - | 12 | 4 | 4 |
| Dropouts | - | - | 4 | 4 | 4 |

**Table 11**. Effect of SkinCera treatment on the percentage subject response to skin oilyness

|  | **Subject visits** | | | | |
| --- | --- | --- | --- | --- | --- |
| **Response** | **Screening** | **Day 1** | **3 weeks** | **6 weeks** | **Follow-up** |
| Oilyness | 48 | 48 | - | - | - |
| Recovery | - | - | 16 | 20 | **28** |
| Improvement | - | - | 8 | 12 | **4** |
| Decrement | - | - | 4 | - | - |
| No response | - | - | 16 | 12 | 12 |
| Dropouts | - | - | 4 | 4 | 4 |

**Table 12**. Effect of SkinCera treatment on the percentage subject response to skin roughness

|  | **Patient visits** | | | | |
| --- | --- | --- | --- | --- | --- |
| **Response** | **Screening** | **Day 1** | **3 weeks** | **6 weeks** | **Follow-up** |
| Roughness | 28 | 28 | - | - | - |
| Recovery | - | - | 12 | 16 | **24** |
| Improvement | - | - | - | - | **4** |
| Decrement | - | - | - | - | - |
| No response | - | - | 16 | 12 | - |
| Dropouts | - | - | - | - | - |

**Table 13.** Summary of vital signs by visit (SkinCera group)

(Safety population)

|  | **Screening** | **Visit 1** | **Visit 2** | **Visit 3** | **Follow-up** |
| --- | --- | --- | --- | --- | --- |
| **Heart rate (bpm)** | | | | | |
| N | 26 | 26 | 23 | 23 | 23 |
| Mean | 77.5 | 77.5 | 77.22 | 78.52 | 78.96 |
| SD | 5.83 | 5.83 | 5.86 | 6.3 | 5.44 |
| Minimum | 65 | 65 | 65 | 65 | 68 |
| Median | 80 | 80 | 78 | 80 | 80 |
| Maximum | 86 | 86 | 88 | 89 | 88 |
| **Systolic blood pressure (mmHg)** | | | | | |
| N | 26 | 26 | 23 | 23 | 23 |
| Mean | 114 | 114 | 113.5 | 113.9 | 113.9 |
| SD | 6.33 | 6.33 | 6.5 | 5.8 | 6.6 |
| Minimum | 100 | 100 | 100 | 110 | 100 |
| Median | 110 | 110 | 110 | 110 | 110 |
| Maximum | 130 | 130 | 130 | 130 | 130 |
| **Diastolic blood pressure (mmHg)** | | | | | |
| N | 26 | 26 | 23 | 23 | 23 |
| Mean | 75 | 75 | 75.2 | 75.7 | 74.4 |
| SD | 5.83 | 5.83 | 5.93 | 5.07 | 5.07 |
| Minimum | 70 | 70 | 70 | 70 | 70 |
| Median | 70 | 70 | 70 | 80 | 70 |
| Maximum | 90 | 90 | 90 | 80 | 80 |

**Table 14**. Summary of vital signs by visit (Placebo group)

(Safety population)

|  | **Screening** | **Visit 1** | **Visit 2** | **Visit 3** | **Follow-up** |
| --- | --- | --- | --- | --- | --- |
| **Heart rate (bpm)** | | | | | |
| N | 25 | 25 | 25 | 17 | 0 |
| Mean | 74.2 | 74.5 | 76.2 | 75.4 | 0 |
| SD | 1.58 | 2.1 | 3.51 | 2.85 | 0 |
| Minimum | 72 | 70 | 68 | 70 | 0 |
| Median | 74 | 75 | 76 | 76 | 0 |
| Maximum | 77 | 78 | 81 | 81 | 0 |
| **Systolic blood pressure (mmHg)** | | | | | |
| N | 25 | 25 | 25 | 17 | 0 |
| Mean | 117 | 118 | 117.4 | 116.2 | 0 |
| SD | 7.37 | 7.52 | 6.5 | 4.9 | 0 |
| Minimum | 110 | 110 | 110 | 110 | 0 |
| Median | 120 | 120 | 120 | 120 | 0 |
| Maximum | 130 | 130 | 130 | 120 | 0 |
| **Diastolic blood pressure (mmHg)** | | | | | |
| N | 25 | 25 | 25 | 17 | 0 |
| Mean | 74.4 | 75.2 | 74 | 80 | 0 |
| SD | 5.06 | 5.1 | 5.0 | 8.7 | 0 |
| Minimum | 70 | 70 | 70 | 70 | 0 |
| Median | 70 | 80 | 70 | 80 | 0 |
| Maximum | 80 | 80 | 80 | 90 | 0 |

**Table 15.** Diagnosis scoring scale for various skin parameters

| **Examination parameter** | **Symptom severity scale** | |
| --- | --- | --- |
| Dryness | No dryness | 0 |
|  | Feeling of dryness after bathing or swimming | 1 |
|  | Dryness associated with itching and redness | 2 |
|  | Dryness and itching interfere with sleep |  |
|  | Deep cracks that may bleed | 3 |
| Whiteheads/blackheads | Normal skin colour without evidence of white heads and black heads | 0 |
|  | Barely visible white heads and black heads | 1 |
|  | Mild white heads and black heads |  |
|  | Moderate white heads and black heads | 2 |
|  | Severe white heads and black heads | 3 |
| Hyperpigmentation | Normal skin colour without evidence of hyperpigmentation | 0 |
|  | Specks of involvement | 1 |
|  | Small patchy areas of involvement < 1.5 cm diameter | 2 |
|  | Patches of involvement > 2 cm diameter |  |
|  | Uniform skin involvement without any clear areas | 3 |
| Redness | No Redness | 0 |
|  | Mild detectable erythema (pink) | 1 |
|  | Dull red clearly distinguishable | 2 |
|  | Deep dark red marked and extensive | 3 |
| Lesions | Less than 10 small lesions (diameter | 0 |
|  | 1-10 to 50 small lesions or less than 10 large lesions (diameter | 1 |
|  | Greater than 50 small lesions or 10 to 50 large lesions and | 2 |
|  | Almost the whole body is covered | 3 |
| Itching | No itching | 0 |
|  | Occasional slight itching | 1 |
|  | Intermittent itching | 2 |
|  | Itching does not interfere sleep |  |
|  | Itching interferes sleep and normal activity | 3 |
| Oilyness | Normal texture | 0 |
|  | Visual methods of assessment (looking in the mirror and looking at the oiliness of blotting paper) | 1 |
|  | Tactile methods of assessment (touching the face and feeling of fingers after stroking or rubbing the face) | 2 |
|  | Sensory feel methods | 3 |
| Roughness | No epidermal roughness | 0 |
|  | Minor epidermal roughness | 1 |
|  | Moderate epidermal roughness but no accentuated skin lines | 2 |
|  | Moderate epidermal roughness with accentuated skin lines |  |
|  | Severe epidermal roughness with deep accentuated skin lines | 3 |
