# Supplementary material for: Potential benefits of oral administration of AMORPHOPHALLUS KONJAC glycosylceramides on skin health – a randomized clinical study
Source: BMC Complement Med Ther. 2020 Jan 31;20:26. doi: 10.1186/s12906-019-2721-3 (PMC7076855; doi:10.1186/s12906-019-2721-3)
Supplement: Supplementary file 2 — Additional file 2. Questionnaire. Diagnosis questionnaire including the symptom scoring scale. [file 12906_2019_2721_MOESM2_ESM.docx]

**Diagnosis scoring scale**

This scale contains questions about how the subject has been diagnosed DURING THE STUDY PERIOD.

| **Examination parameter** | **Symptom severity scale** | |
| --- | --- | --- |
| Dryness | No dryness | 0 |
|  | Feeling of dryness after bathing or swimming | 1 |
|  | Dryness associated with itching and redness | 2 |
|  | Dryness and itching interfere with sleep |  |
|  | Deep cracks that may bleed | 3 |
| Whiteheads/blackheads | Normal skin colour without evidence of white heads and black heads | 0 |
|  | Barely visible white heads and black heads | 1 |
|  | Mild white heads and black heads |  |
|  | Moderate white heads and black heads | 2 |
|  | Severe white heads and black heads | 3 |
| Hyperpigmentation | Normal skin colour without evidence of hyperpigmentation | 0 |
|  | Specks of involvement | 1 |
|  | Small patchy areas of involvement < 1.5 cm diameter | 2 |
|  | Patches of involvement > 2 cm diameter |  |
|  | Uniform skin involvement without any clear areas | 3 |
| Redness | No Redness | 0 |
|  | Mild detectable erythema (pink) | 1 |
|  | Dull red clearly distinguishable | 2 |
|  | Deep dark red marked and extensive | 3 |
| Lesions | Less than 10 small lesions (diameter | 0 |
|  | 1-10 to 50 small lesions or less than 10 large lesions (diameter | 1 |
|  | Greater than 50 small lesions or 10 to 50 large lesions and | 2 |
|  | Almost the whole body is covered | 3 |
| Itching | No itching | 0 |
|  | Occasional slight itching | 1 |
|  | Intermittent itching | 2 |
|  | Itching does not interfere sleep |  |
|  | Itching interferes sleep and normal activity | 3 |
| Oilyness | Normal texture | 0 |
|  | Visual methods of assessment (looking in the mirror and looking at the oiliness of blotting paper) | 1 |
|  | Tactile methods of assessment (touching the face and feeling of fingers after stroking or rubbing the face) | 2 |
|  | Sensory feel methods | 3 |
| Roughness | No epidermal roughness | 0 |
|  | Minor epidermal roughness | 1 |
|  | Moderate epidermal roughness but no accentuated skin lines | 2 |
|  | Moderate epidermal roughness with accentuated skin lines |  |
|  | Severe epidermal roughness with deep accentuated skin lines | 3 |
