# Supplementary material for: Potential benefits of oral administration of AMORPHOPHALLUS KONJAC glycosylceramides on skin health – a randomized clinical study
Source: BMC Complement Med Ther. 2020 Jan 31;20:26. doi: 10.1186/s12906-019-2721-3 (PMC7076855; doi:10.1186/s12906-019-2721-3)
Supplement: Supplementary file 3 — Additional file 3. Characterization of constituents in SkinCera. Details of other constituents of SkinCera apart from glycosylceramides 1–4. [file 12906_2019_2721_MOESM3_ESM.docx]

**Characterization of constituents in SkinCera**

**Table 1.** Proximate and chemical analysis of SkinCera.

| **Constituents** | **Value (%)** | **RSD** |
| --- | --- | --- |
| Polyphenols^1^ | 9.1 ± 0.38 | 4.178 |
| Flavonoids^2^ | 2.0 ± 0.03 | 1.580 |
| Protein^3^ | 14.8 ± 0.12 | 0.819 |
| Total carotenoids^4^ | 0.3 ± 0.01 | 5.051 |

The values are expressed as Mean ± SD., n= 3

Total phenols were determined by Folin Ciocalteu reagent as described by McDonald et al., 2001. For the determination of total flavonoid content aluminium chloride colorimetric method described by Chang et al., 2002 was employed. The determination of the total protein content is based on measurement of total nitrogen by Kjeldahl apparatus.

**References**

1. S McDonald, PD Prenzler, M Antolovich, K Robards. Phenolic content and antioxidant activity of olive extracts Food Chem, 73 (2001), 73-84.
2. CC Chang, MH Yang, HM Wen, JC Chern. Estimation of total flavonoid content in propolis by two complementary colorimetric methods. J Food Drug Anal, 10 (2002), 178-182.
3. Joanna M. Lynch and david M. Barbano. Kjeldahl Nitrogen Analysis as a Reference Method for Protein Determination in Dairy Products. Food Composition and Additives. 82 (6), 1999, 1389-1398.
4. Content of Total Carotenoids. The United States Pharmacopoeial Convention.2013.

**Qualitative analysis of sphingolipids**

Qualitative analysis of sphingolipids was performed in LC-ESI-MS/MS-8040 (Shimadzu) triple quadrapole mass spectrometer. This instrument was equipped with electrospray ionisation (ESI) source. The mass spectrometer chromatogram conditions as follows: nebulizer gas flow, 3 L/min; drying gas flow, 13 L/min; desolvation line (DL) temperature, 250 °C; heat block temperature, 400 °C in ESI source. Other parameters were tuned automatically. The separation was carried out in Kinetex using a XB-C18 column (150 mm×2.1 mm, 2.6 µm) at a flow rate of 0.3 mL/min in ESI source. The mobile phase consists of gradient elution with a low-pressure gradient using 0.1% formic acid: methanol (A:B): with a flow rate of 1.0 ml/min and the injection volume of 5 μl. All solutions were degassed and filtered through 0.2 μm pore size filter. The column was maintained at 40 °C throughout analysis. Methanol used as a diluent for assay by HPLC analysis and the total run time was 15 min. The separations were effected by gradient program as follows: from 0.01 to 7 min, solution B followed a linear change from 0% to 100%; from 7 to 10 min, B was isocratic at 100%; from 10 to 12 min, B linearly changed from 100% to 0% and from 12 to 15.

**Figure 1** shows the mass chromatogram of sphingolipids in SkinCera. The ceramides such as **C18** (m/z 566.6 at RT 6.788 min), **C24** (m/z 650.8 at RT 7.172 min), **C16** (m/z 538.6 at RT 9.485 min) and glucosylceramide **C16** (m/z 700.7 at RT 9.315 min) were identified by SIM mode (**Figure 2**). This data is accordance with other previous reports (Zhang et al., 2013).


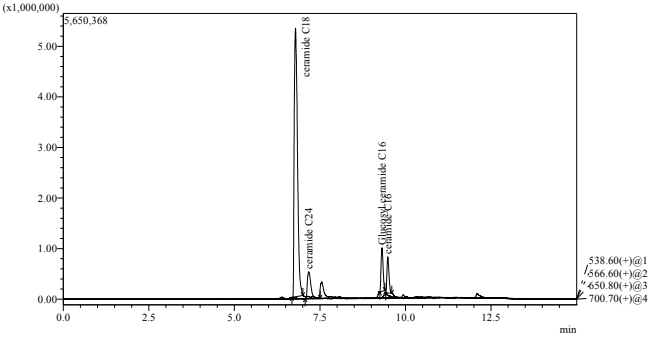


**Figure 1.** Selected Ion monitoring (SIM) chromatogram of Sphingolipids in Konjac by LC-MS/MS.


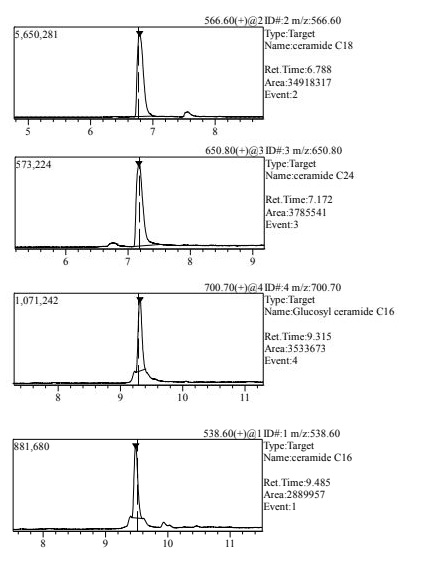


**Figure 2.** Qualitative chromatogram of sphingolipids in SkinCera by LC-MS/MS

**Table 2** shows the other Sphingolipids presence such as SPC, DimetSPH, TrimetSPH, PhytoSPH and SPA. This data is pertaining to other previous reports Scherer et al., 2010.

**Table 2.** Other Sphingolipid Mass profile in SkinCera by positive ion ESI mode.

| **Sphingolipids** | **Precursor (M + H)^+^**  **m/z** | **Collision**  **Energy** | **Fragment** |
| --- | --- | --- | --- |
| SPC | 465.2 | -50 | 184.20 |
| DimetSPH | 328.2 | -22 | 280.5 |
| TrimetSPH | 342.1 | -28 | 60.0 |
| PhytoSPH | 318.4 | -16 | 282.4 |
| SPA | 302.3 | -16 | 284.30 |

Where, SPC- Sphingosylphosphorylcholine, DimetSPH- Dimethyl Sphingosine, TrimetSPH- Trimethyl Sphingosine, PhySPH- Phyto Sphingosine, SPA-Spinganine.

Further, qualitative analysis of SkinCera revealed the presence of cinnamic acid, riboflavin, vanillic acid, serotinin, coumaryl serotinin and thiamine (**Figure 3**).


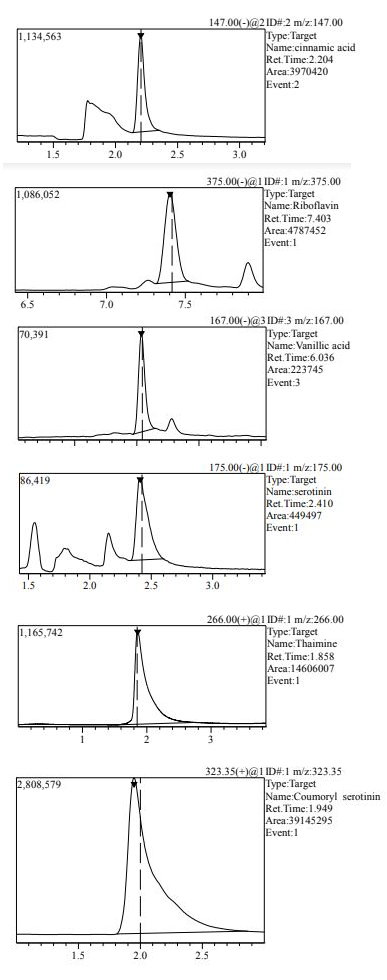


**Figure 3.** Qualitative chromatogram of SkinCera by positive ion ESI SIM mode
