# Supplementary material for: Potential benefits of oral administration of AMORPHOPHALLUS KONJAC glycosylceramides on skin health – a randomized clinical study
Source: BMC Complement Med Ther. 2020 Jan 31;20:26. doi: 10.1186/s12906-019-2721-3 (PMC7076855; doi:10.1186/s12906-019-2721-3)
Supplement: Supplementary file 4 — Additional file 4. Sample size calculation. [file 12906_2019_2721_MOESM4_ESM.docx]

**Sample size calculation**

The sample size calculation was based on difference b/w mean scores of 2 treatments are considered to be medically relevant. Assuming a common SD of 1.8 for the number of Moisturizing effect and reducing the wrinkle formation e.t.c. at the end of treatment, 20 per group would be sufficient to detect a difference of 1.63 in mean difference score b/w the 2 treatment with power of 80% and a 0.05. 2-sided level of significance.

**R-Program:**

power.t.test(n=NULL, delta=1.63, sd=1.8, sig.level=0.05, power=0.80,

type="two.sample", alternative="two.sided")

Two-sample t test power calculation

n = 20.14987

delta = 1.63

sd = 1.8

sig.level = 0.05

power = 0.8

alternative = two.sided

NOTE: n is number in *each* group

A total of *N* number of subjects are required at each Treatment group in the end of the study with all the data being complete for analysis, but a proportion (*q*) are expected to drop out before the study ends. In this case, the following total number of subjects (*N1*) would have to be enrolled to ensure that the final sample size (*N*) in each Treatment group is:

$$N1=\frac{N}{1-q}=\frac{40}{\left( 1-0.10 \right)}=44$$

Where *q* is the proportion of attrition and is generally 10% in this type of studies.

Note: The proportion of eligible subjects who will refuse to participate (drop out) or provide the inadequate information will be unknown at the beginning of the study. Approximate estimates is often possible using information from similar studies.
